# Suppression of kidney fibrosis by cGMP-dependent protein kinase I

**DOI:** 10.1186/2050-6511-14-S1-P61

**Published:** 2013-08-29

**Authors:** Elisabeth Schinner, Andrea Schramm, Frieder Kees, Franz Hofmann, Jens Schlossmann

**Affiliations:** 1 Pharmakologie und Toxikologie, Institut für Pharmazie, Universität Regensburg, Germany; 2 Carvas-Zentrum, TU München, Germany

## Background

cGMP is synthesized via nitric oxide- or natriuretic peptide-stimulated guanylyl cyclases and exhibits pleiotropic regulatory functions also in the kidney. Both isoforms of cGKI (α, β) have been detected in arterioles, mesangium and within the cortical interstitium. In contrast to cGKIα, the β-isoform was not detected in the juxtaglomerular apparatus and in medullary fibroblasts.

The aim of this study was to examine the function of cGKI in the renal interstitium, emphasizing a functional differentiation of both isoforms. Interstitium fibroblasts play a prominent role in interstitial fibrosis. Accordingly, cGKI may also be involved in this pathophysiological process.

## Results

Kidney fibrosis was induced by unilateral ureter obstruction (UUO). We treated αSM-rescue (expressing cGKIα only in smooth muscle under the control of the SM22 promotor with a cGKI-KO background), cGKI-KO mice (expressing no cGKI) and wt mice with YC-1 (sGC stimulator) which increases cGMP concentration.

Administration of YC-1 showed significantly antifibrotic effects in wt-, but not in αSM-rescue- and cGKI-KO mice, especially regarding the fibrosis marker Col1a1, TGFβ and fibronectin. Thereby cGKIα was activated by YC-1 which phosphorylates RhoA and inhibits in turn the profibrotic RhoA/ROCK pathway.

## Conclusion

Our results indicate that cGMP/cGKIα acts via RhoA/ROCK, as an important suppressor of kidney fibrosis.

